# Exploring the Bidirectional Associations Between Short or Long Sleep Duration and Lower Cognitive Function: A 7-Year Cohort Study in China

**DOI:** 10.3389/fnagi.2021.727763

**Published:** 2021-10-06

**Authors:** Jianian Hua, Sheng Zhuang, Yueping Shen, Xiang Tang, Hongpeng Sun, Qi Fang

**Affiliations:** ^1^Department of Neurology, The First Affiliated Hospital of Soochow University, Suzhou, China; ^2^Medical College of Soochow University, Suzhou, China; ^3^Department of Neurology, Suzhou Clinical Research Center of Neurological Disease, The Second Affiliated Hospital of Soochow University, Suzhou, China; ^4^Department of Epidemiology and Biostatistics, School of Public Health, Medical College of Soochow University, Suzhou, China; ^5^Department of Child Health, School of Public Health, Medical College of Soochow University, Suzhou, China

**Keywords:** sleep duration, sleep, cognitive function, cognition, dementia, cross-lagged models, cohort study, aging

## Abstract

**Background:** Sleep duration is linked to cognitive function, but whether short or prolonged sleep duration results from impaired cognition or vice versa has been controversial in previous studies. We aimed to investigate the bidirectional association between sleep duration and cognitive function in older Chinese participants.

**Methods:** Data were obtained from a nationally representative study conducted in China. A total of 7984 participants aged 45 years or older were assessed at baseline between June 2011 and March 2012 (Wave 1), 2013 (Wave 2), 2015 (Wave 3), and 2018 (Wave 4). Nocturnal sleep duration was evaluated using interviews. Cognitive function was examined via assessments of global cognition, including episodic memory, visuospatial construction, calculation, orientation and attention capacity. Latent growth models and cross-lagged models were used to assess the bidirectional association between sleep duration and cognitive function.

**Results:** Among the 7,984 participants who were followed in the four waves of the study, the baseline mean (SD) age was 64.7 (8.4) years, 3862 (48.4%) were male, and 6453 (80.7%) lived in rural areas. Latent growth models showed that both sleep duration and global cognition worsened over time. Cross-lagged models indicated that short or long sleep duration in the previous wave was associated with lower global cognition in the subsequent wave (standardized β = −0.066; 95% CI: −0.073, −0.059; *P* < 0.001; Wave 1 to 2) and that lower global cognition in the previous wave was associated with short or long sleep duration in the subsequent wave (standardized β = −0.106; 95% CI: −0.116, −0.096; *P* < 0.001; Wave 1 to 2).

**Conclusion:** There was a bidirectional association between sleep duration and cognitive function, with lower cognitive function having a stronger association with long or short sleep duration than the reverse relationship. Global cognition was likely the major driver in these reciprocal associations.

## Introduction

One fourth of the global population was 45 years or older in 2015 (United Nations Department of Economic and Social Affairs Population Division, 2020). As people continue to age worldwide, the number of middle-aged and older adults with cognitive impairment and dementia is increasing rapidly (Report, 2020). Dementia is a neurodegenerative disorder that is characterized by cognitive decline (McKhann et al., 2011). Even in the general population, declined cognitive function is associated with poorer health and quality of life, impairments in functional abilities, increased medical costs, and development of dementia (Plassman et al., 2010; Jekel et al., 2015). Promoting successful cognitive aging is of great importance for global health. Exploring the relationships between modifiable risk factors and cognitive function is essential for developing intervention strategies aimed at maintaining brain health into older age (Corley et al., 2018).

Aging also leads to changes in sleep patterns, such as shorter sleep duration and longer sleep latency. Short and long sleep durations are associated with various diseases and conditions, such as metabolic syndromes and stroke (Itani et al., 2017; Jike et al., 2018; Hua et al., 2021), all of which impair quality of life. Moreover, sleep plays an important role in cognitive function, with solid evidence suggesting an inverted “U-shaped” association between sleep duration and cognitive scores; that is, both short and long sleep durations are correlated with lower cognitive function. This theory was suggested by cross-sectional studies, longitudinal studies, and a Mendelian randomization study (Henry et al., 2019). Our previous study linked changes in sleep duration from the optimal sleep pattern to lower cognition (Hua et al., 2020b). Sleep restrictions in laboratories and shift-work jobs are also detrimental to cognition in the short term (Machi et al., 2012). One mechanism that has been proposed to explain these phenomena relies on the triggering of Alzheimer’s disease (AD)-related pathologies, which leads to cognitive decline (Cox et al., 2019).

Numerous studies have reported the effects of sleep on cognition. Recent evidence showed that AD biomarkers in subjects with decreased cognitive function could trigger sleep dysregulation (Zhang et al., 2019; Liguori et al., 2020). Epidemiological studies have reported that sleep disorders occurred in patients with AD-related mild cognitive impairment (MCI) and that their frequency increased with disease progression (Guarnieri et al., 2012; Baillon et al., 2019). Sleep disorders included sleep-disordered breathing, insomnia, and rapid eye movement (REM) behavior disorder. People with self-reported cognitive decline had lower sleep efficiency and increased wakefulness (Lauriola et al., 2017). Individuals at different stages of dementia demonstrated different sleep architectures, such as less REM sleep, more stage 1 non-REM sleep, and less stage 3 non-REM sleep (Vitiello et al., 1990; Liguori et al., 2020). However, the samples of the previous studies were small. Moreover, some of these studies did not consider nocturnal sleep duration as an outcome variable, and most of them were based on a cross-sectional design, which provided only a snapshot of the association at a certain time point and introduced a more substantial bias.

To the best of our knowledge, no population-based studies have examined cognitive function as a predictor of nocturnal sleep duration. Furthermore, no studies have explored the bidirectional associations between sleep duration and cognitive function. It is far from clear whether sleep duration is a risk factor for lower cognition or whether sleep duration is a marker of cognitive decline.

To address the current gap, we used the China Health and Retirement Longitudinal Study (CHARLS), a nationally representative cohort, to investigate sleep duration not only as a predictor but also as a consequence of cognitive function. The aims of our study were to (1) evaluate whether a bidirectional association existed between short or long sleep duration and lower cognitive function; (2) determine how the two factors were associated with each other over time; and (3) identify the driving factor.

## Materials and Methods

### Study Population

China Health and Retirement Longitudinal Study is a community-based and nationally representative cohort study in China and a sister study of the Health and Retirement Study (HRS) in United States (Zhao et al., 2014). Wave 1 (baseline) of the CHARLS occurred in 2011 and included approximately 18,000 randomly selected Chinese residents who were enrolled via multistage probability sampling. Follow-up surveys were performed every 2 years. The present study used CHARLS data from Wave 1 (2011), Wave 2 (2013), Wave 3 (2015), and Wave 4 (2018).

**FIGURE 1:**
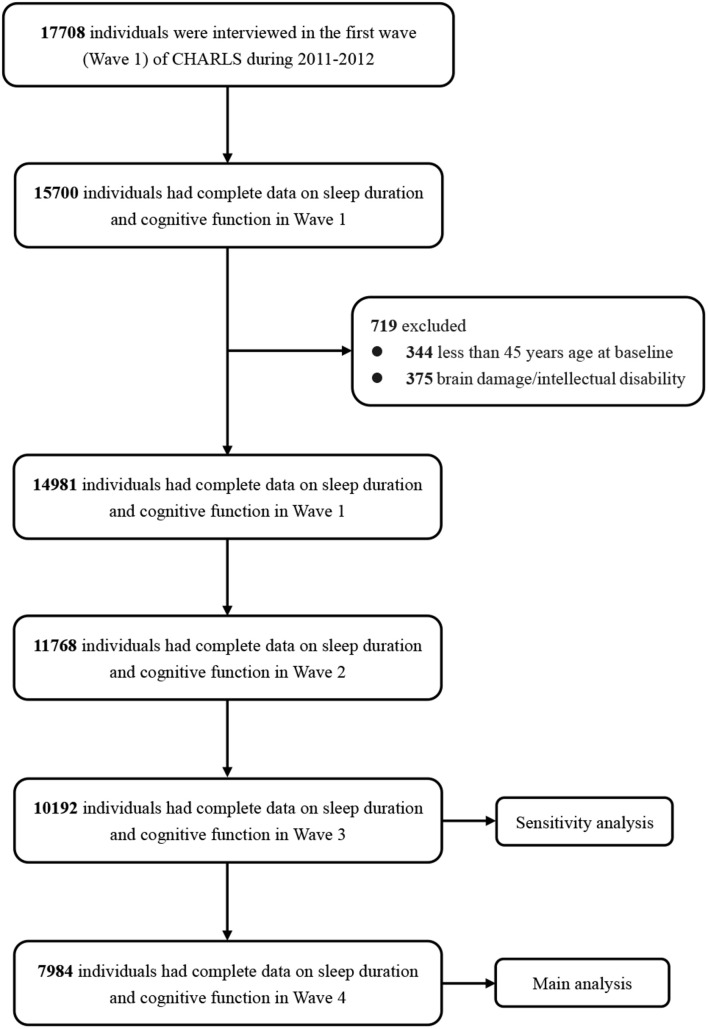
Flowchart of participant selection.

Figure 1 shows the flow chart of the sample selection and exclusion criteria. Data on complete cognitive function tests and sleep duration in Wave 1 were available for 15,700 individuals. At baseline, 344 individuals aged less than 45 years and 375 individuals who reported brain damage or intellectual disability were excluded, as were individuals with missing data on sleep duration or cognitive function in the follow-up waves. Among the 14,981 participants with complete baseline data, 11,768 (78.6%) had complete data on sleep duration and cognitive function in Wave 2, 10,192 (68.1%) in Wave 3, and 7984 (50.1%) in Wave 4.

### Ethics Approval

Each respondent who agreed to participate in the survey was asked to sign two copies of the informed consent form. The details are shown in Supplementary Text 1.

### Sleep Duration

The sleep duration measured for this study was nocturnal sleep duration. It was assessed by asking the participants the following question in all four waves: “During the last month, how many hours of actual sleep did you get at night on average”? Participants reported sleep duration to the nearest 0.5 h.

### Cognitive Assessment

Cognitive assessment was performed in all four waves and included three domains: episodic memory; visuospatial construction, as measured using the figure drawing test; and orientation, attention and calculation, as measured using the CHARLS version telephone interview of cognitive status (TICS) (Meng et al., 2019). The participant’s cognitive function was represented by the score of global cognition, which resulted from the sum of these three scores. The global cognition score ranged from 0 to 21. The components of global cognition are presented in the Supplementary Text 2.

### Covariates

The following covariates were included: baseline age (in years); sex; and baseline living area (urban or rural) (Zheng et al., 2018). None of our participants had missing covariate data on age, sex, or living area.

### Statistical Analysis

In the main analysis, the sleep duration value for subjects who slept less than 4 h or longer than 10 h was defined as “1.” The sleep duration value of subjects who slept between 4 and 10 h was defined as “0.” The selection of the cut-off point was based on the following evidence. First, our previous CHARLS study reported a cross-sectional “inverted-U”-shaped association between sleep duration and cognition. The optimal sleep duration for cognitive function was approximately 7 h (Hua et al., 2020b). Second, a previous article on CHARLS reported that less than 4 h or more than 10 h of sleep accelerated cognitive decline during a follow-up of 4 years compared with a 7 h of sleep (Ma et al., 2020). Other cut-off points (<5 h, <6 h, >8 h, or >9 h) did not accelerate cognitive decline. The sensitivity analysis examined two other cut-off points, namely, 5–9 h and 6–8 h. Third, few people had a long sleep duration. This small sample size might reduce the statistical power. In sensitivity analysis, short sleep duration and long sleep duration were examined separately.

Our previous study defined people with “excessive change” as subjects who slept for a short duration in the previous wave and a long duration in the next wave, or vice versa. “Excessive change” was strongly associated with lower cognitive function (Supplementary Figure 1; Hua et al., 2020b). Because we defined both short and long sleep durations as “1” in this study, “excessive change” could not be observed. Fortunately, none of the 7,984 participants exhibited “excessive change” in the dataset for the main analysis. In other sensitivity analysis subgroups, individuals with “excessive change” were excluded.

In the main text, two models were used to explore the associations. First, the separate latent growth models showed the trajectories of each variable. The combined latent growth models were used to investigate whether a bidirectional association existed between sleep duration and global cognition. Second, the cross-lagged models determined the effect of a variable in the previous wave on another variable in the subsequent wave. Our main conclusions were drawn from the cross-lagged models.

**FIGURE 2:**
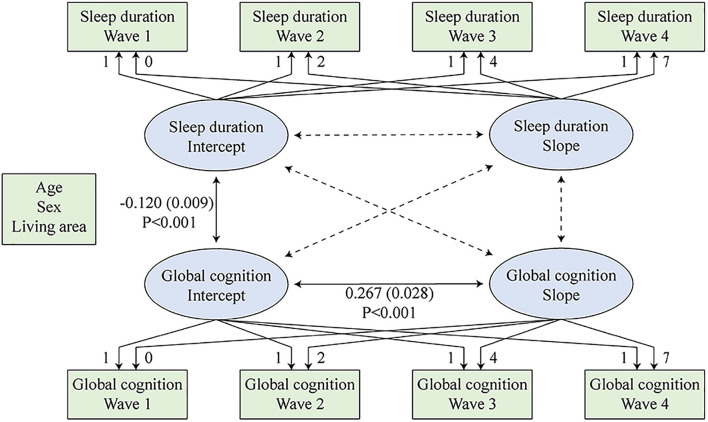
Combined latent growth models of sleep duration and global cognition. Sleep duration intercept, sleep duration slope, global cognition intercept, and global cognition slope were regressed on the covariates simultaneously.

The latent growth models consisted of two stages. In the first stage, the separate latent growth models examined the trends of the two variables separately. Since both global cognition and sleep duration may decline due to aging, they were hypothesized to follow a pattern of change, which can be described by intercept (baseline) and slope (change). The linear latent growth models were used. Supplementary Table 2 provides a description of the intraindividual/within-person mean for the repeated measure (intercept/baseline), interindividual/between-person variance in the intercept (intercept variance), intraindividual mean change (slope), and interindividual difference in intraindividual mean change (slope variance). We also considered quadratic latent growth models. The addition of the quadratic factors improved models fit. However, either the quadratic slope variance of global cognition or the quadratic slope of sleep duration value was not statistically significant (Supplementary Table 3). Thus, we elected to use the linear models. For the combined models, the conclusions remained similar after the addition of quadratic terms. The second stage combined the above two models into one model, and this combined model examined correlations between the sleep duration intercept, sleep duration slope, global cognition intercept, and global cognition slope (McArdle, 2009; Zheng et al., 2018). The standardized correlation value is the correlation of the variables’ *z* scores. Equations in the combined model were estimated simultaneously while controlling for covariates. The construction of the combined latent growth model is shown in Figure 2.

**FIGURE 3:**
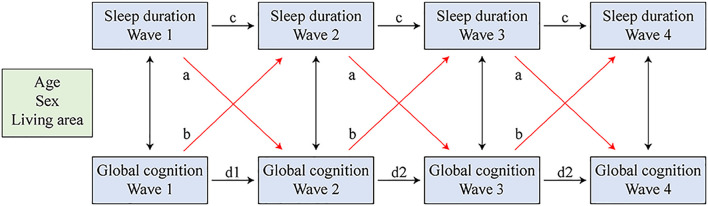
Cross-lagged models of sleep duration and global cognition. Sleep duration and global cognition in all four waves were regressed on the covariates simultaneously. Paths that were constrained equal were marked with the same letter.

Cross-lagged models studied the effect of one variable in the previous wave on the other variable in the subsequent wave (Figure 3). Sleep duration at each time point was regressed on sleep duration and global cognition at the prior time point. Similarly, global cognition at each time point was regressed on sleep duration and global cognition of the prior time point. Sleep duration and global cognition measured in the same wave were correlated. All equations were estimated simultaneously while controlling for covariates. The standardized coefficients are provided as a common metric of standard deviation (SD) units. To achieve a more parsimonious model, we constrained the cross paths to equality across waves. We used a weighted least squares estimator (WLSMV) that employed regression for categorical outcomes (Kamin and Lang, 2020).

The latent growth models and cross-lagged models were examined using model fit statistics, including the comparative fit index (CFI), Tucker-Lewis index (TLI), and root mean squared error of approximation (RMSEA). A CFI/TLI value over 0.90 indicates a good fit, and values above 0.95 indicate an excellent fit. We used a sandwich estimator for the standard errors, which is robust for non-normality (Ke-Hai Yuan, 2000).

Statistical analyses were 2-sided, with α = 0.05 being the threshold for statistical significance. Structural equation modeling was performed using Mplus 8.3 (Muthén and Muthén) (Muthén, 2016; Kamin and Lang, 2020). Other analyses were performed using SAS version 9.4 (SAS Institute Inc., Cary, NC, United States). The pooled effects in the sensitivity analysis were calculated using Stata 15.1 (Stata Corp., College Station, TX, United States).

### Sensitivity Analysis

Two widely used and rigorous analyses were performed to corroborate the findings from the latent growth models. Cox regression was used to assess the associations between baseline global cognition and follow-up sleep duration. All 7,984 participants were divided into quantiles based on their global cognition scores at baseline. Endpoints in Cox models were defined as a sleep duration of 4 or 10 h. Kaplan–Meier curves, the log-rank test, the Wilcoxon test and the −2 log test were used to compare the cumulative risk of events over quantiles of baseline global cognition. Multivariate Cox proportional hazards regression was used to estimate hazard ratios and 95% confidence intervals. The generalized estimating equation (GEE) approach was used to analyze the associations between baseline sleep duration and follow-up global cognition score. The interaction of the time variable with sleep duration was used to examine whether rates of cognitive decline varied by baseline sleep duration.

**FIGURE 4:**
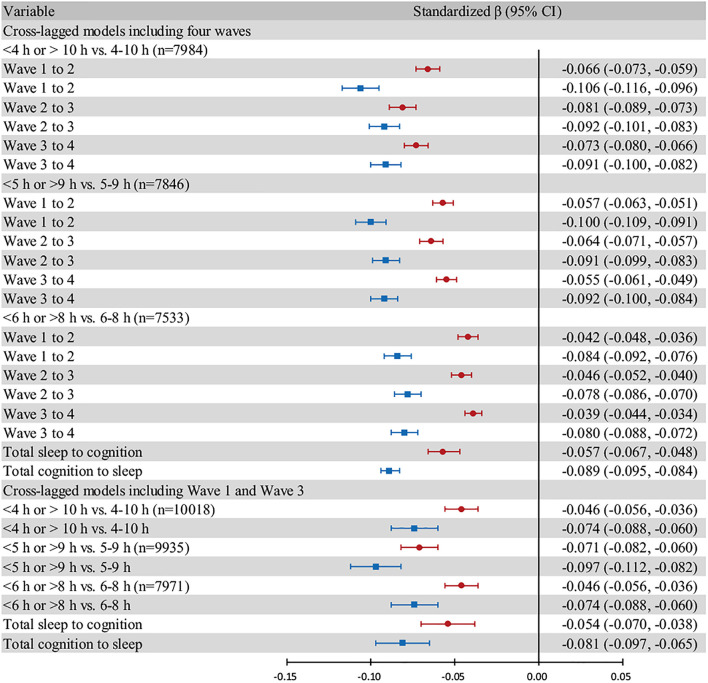
Forest plot based on different cut-off points and models. Individuals who exhibited “excessive change” are excluded. All models are adjusted for age, sex, and living area. The red bars show the effect of sleep duration in the previous wave on global cognition in the next wave. The blue bars show the effect of global cognition in the previous wave on sleep duration in the next wave. The “total” group represent the pooled effect. Error bars show the standardized beta coefficient (points) and 95% CIs (whiskers).

We were able to achieve better model fitness when including only Wave 1 and Wave 3 in cross-lagged models for the following reasons. First, global cognition scores in Wave 1 and Wave 3 had similar standard deviations, which were more homogeneous (Table 1). Second, the time interval between Wave 1 and Wave 3 was longer, during which time more cognitive decline could be observed. Therefore, we performed a cross-lagged model for Wave 1 and Wave 3 as a sensitivity analysis. Sleep duration and global cognition in Wave 1 and Wave 3 were controlled for age, sex, and living area simultaneously (Supplementary Tables 8, 9 and Figure 4).

**TABLE 1:** Characteristics of the study population at each wave*.

	**Wave 1**	**Wave 2**	**Wave 3**	**Wave 4**
*N*	14981	11768	10192	7984
Age (years)	59.4 ± 9.7	60.8 ± 9.1	62.4 ± 8.8	64.7 ± 8.4
Male	7169 (47.9)	5662 (48.1)	5512 (48.2)	3862 (48.4)
Living area				
Rural	11591 (76.3)	9276 (51.9)	7925 (80.1)	6453 (80.7)
Urban	3550 (23.7)	2492 (21.0)	2267 (19.9)	1531 (19.3)
Global cognition score	10.4 ± 4.4	10.6 ± 4.3	10.2 ± 4.3	9.7 ± 4.9
Sleep duration				
4–10 h	13779 (91.3)	10658 (90.6)	9276 (91.0)	7080 (88.6)
<4 h	1191 (8.0)	1028 (8.7)	856 (8.4)	810 (10.2)
>10 h	11 (0.7)	82 (0.7)	60 (0.6)	94 (1.2)

*The results are presented as the mean ± SD or *n* (%).

The cross-lagged models including Wave 1 and Wave 3 indicated an excellent fit even after controlling for more demographic and health covariates. The covariates were assessed at baseline (Wave 1) by interview and included age, sex, living area, educational level, marital status, current smoking, current drinking, hypertension, dyslipidaemia, diabetes, cancer, lung diseases, heart problems, depression (with a Center for Epidemiological Survey-Depression Scale score not less than 12 points), and the number of instrumental activities of daily living (IADLs). Sleep duration and global cognition in Wave 1 and Wave 3 were controlled for the covariates simultaneously. Three individuals with missing data on covariates were excluded from this analysis.

In the forest plot (Figure 4), we chose three cut-off points, namely, “4–10 h,” “5–9 h,” and “6–8 h”, for moderate sleep duration and assessed cross-lagged models including all four waves and cross-lagged models including only Wave 1 and Wave 3. We estimated the pooled beta coefficient and 95% CI using random-effect models.

As a sensitivity analysis, we examined the bidirectional relationship between short sleep duration and global cognition and between long sleep duration and global cognition. Individuals with a long sleep duration were excluded from the analysis of short sleep duration, and those with a short sleep duration were excluded from the analysis of long sleep duration. Then, none of them had an “excessive change.” In another sensitivity analysis, we excluded 96 participants with the self-reported memory-related problems at baseline. The cross-lagged models across the four waves were used to study the above associations. All three cut-off points were examined.

## Results

At baseline, the mean age was 59.4 ± 9.7 years, 47.9% of the participants were male, and 76.3% of the participants were living in a rural area (Table 1). The mean global cognition score of all participants at baseline was 10.4 ± 4.4. The sleep duration of most of the participants was between 4 and 10 h: 8.0% of the individuals slept less than 4 h, and 0.7% slept more than 10 h. During follow-up, more people slept less than 4 h or more than 10 h. The mean global cognition score decreased over time. Supplementary Table 1 provides further data on loss to follow-up. People who were lost to follow-up were more likely to be older and live in urban areas. There was no significant difference in sex.

### Latent Growth Models

In the first stage, we separately calculated the mean baseline value (intercept) and the rate of change (slope) for sleep duration and global cognition using the separate latent growth models. For sleep duration, the intercept was 0.071 (95% CI: 0.048, 0.094; *P* < 0.001), and the average worsening per year (slope) was 0.006 (95% CI: 0.006, 0.006; *P* < 0.001). This model exhibited an excellent fit (CFI = 0.989, TLI = 0.987, RMSEA = 0.018). For global cognition, the intercept was 11.362 (95% CI: 11.321, 11.403; *P* < 0.001), and the change rate per year (slope) was −0.201 (95% CI: −0.207, −0.195; *P* < 0.001). This model also showed an excellent fit (CFI = 0.982, TLI = 0.978, RMSEA = 0.084). The variances of all variables were statistically significant, which indicated individual differences in the intercept and slope for sleep duration and global cognition. Hence, the sleep duration value and global cognition score worsened over time (Supplementary Table 2).

In the second stage, the intercept and slope of sleep duration and global cognition were considered together using a simultaneous equation (Figure 2 and Table 2). In Figure 2, the significant and non-significant paths are drawn as solid and dotted lines, respectively. The overall model fit was excellent (CFI = 0.985, TLI = 0.974, RMSEA = 0.034). A worse baseline sleep duration value was significantly associated with a lower baseline global cognition score (unstandardized *r* = −0.120; 95% CI: −0.129, −0.111; *P* < 0.001). A higher baseline global cognition score was associated with a slower decline rate in global cognition (unstandardized *r* = 0.267; 95% CI: 0.239, 0.295; *P* < 0.001). We also observed a trend that a faster worsening rate of sleep duration value was associated with a deeper decline rate of global cognition (unstandardized *r* = −0.001; 95% CI: −0.001, −0.001; *P* = 0.059; standardized *r* = −0.216; 95% CI: −0.336, −0.096; *P* = 0.072). However, the association was not statistically significant. The results for confounders are shown in Supplementary Table 4.

**TABLE 2:** Correlations between the trajectories using the combined latent growth models.

	***r* (95% CI)**	***P*-value**
**Standardized**		
Sleep duration intercept – Sleep duration slope	−0.120 (−0.181, −0.059)	0.050
Global cognition intercept – Global cognition slope	0.714 (0.533, 0.895)	<0.001
Sleep duration intercept – Global cognition intercept	−0.319 (−0.341, −0.297)	<0.001
Sleep duration slope – Global cognition slope	−0.216 (−0.336, −0.096)	0.072
Sleep duration intercept – Global cognition slope	0.027 (−0.039, 0.093)	0.683
Global cognition intercept – Sleep duration slope	−0.069 (−0.109, −0.029)	0.081
**Unstandardized**		
Sleep duration intercept – Sleep duration slope	0.000 (0.000, 0.000)	0.090
Global cognition intercept – Global cognition slope	0.267 (0.239, 0.295)	<0.001
Sleep duration intercept – Global cognition intercept	−0.120 (−0.129, −0.111)	<0.001
Sleep duration slope – Global cognition slope	−0.001 (−0.001, −0.001)	0.059
Sleep duration intercept – Global cognition slope	0.001 (0.000, 0.001)	0.683
Global cognition intercept – Sleep duration slope	−0.003 (−0.005, −0.001)	0.079

*r, correlation coefficients.*

### Cross-Lagged Models

The combined latent growth models reflected only the existence and direction of the bidirectional association and did not determine whether the changes in global cognition were due to sleep duration, or vice versa. Therefore, we evaluated cross-lagged models to explore the extent to which sleep duration and global cognition predicted one another over time (Figure 3; paths constrained to be equal are marked with the same letter). Autoregressive paths (c and d, Figure 3) accounted for the stability of the measures, and the cross-lagged paths (a and b) indicated the effect of one variable in the previous wave on the other variable in the subsequent wave.

The cross-lagged models including all four waves fit well (CFI = 0.962, TLI = 0.906, RMSEA = 0.059). Sleep duration in one wave was associated with sleep duration in the subsequent wave (unstandardized β = 0.737; 95% CI: 0.718, 0.756; *P* < 0.001), and global cognition in one wave was associated with global cognition in the next wave (unstandardized β = 0.602; 95% CI: 0.593, 0.611; *P* < 0.001; Table 3).

**TABLE 3:** Cross-lagged models across all four waves*.

	**Wave 1 to 2**	**Wave 2 to 3**	**Wave 3 to 4**
**Standardized β (95% CI)**			
Sleep duration to global cognition	−0.066 (−0.073, −0.059)	−0.081 (−0.089, −0.073)	−0.073 (−0.080, −0.066)
Global cognition to sleep duration	−0.106 (−0.116, −0.096)	−0.092 (−0.092, −0.092)	−0.091 (−1.000, −0.082)
Sleep duration to sleep duration	0.580 (0.569, 0.591)	0.006 (−0.008, 0.020)	0.708 (0.602, 0.814)
Global cognition to sleep duration	0.624 (0.616, 0.632)	0.807 (0.799, 0.815)	0.675 (0.668, 0.682)
**Unstandardized β (95% CI)**			
Sleep duration to global cognition	−0.249 (−0.275, −0.223)	−0.249 (−0.275, −0.223)	−0.249 (−0.275, −0.223)
Global cognition to sleep duration	−0.034 (−0.037, −0.031)	−0.034 (−0.037, −0.031)	−0.034 (−0.037, −0.031)
Sleep duration to sleep duration	0.737 (0.718, 0.756)	0.737 (0.718, 0.756)	0.737 (0.718, 0.756)
Global cognition to sleep duration	0.602 (0.593, 0.611)	0.602 (0.593, 0.611)	0.602 (0.593, 0.611)

*P < 0.001 for all values.

Short or long sleep duration in the previous wave was associated with lower global cognition in the ensuing wave (unstandardized β = −0.249; 95% CI: −0.275, −0.223; *P* < 0.001), and lower global cognition in the previous wave was associated with worse (short or long) sleep duration in the subsequent wave (unstandardized β = −0.034; 95% CI: −0.037, −0.031; *P* < 0.001). The results for confounders are shown in Supplementary Table 5.

Comparisons of standardized beta coefficients were comparable and revealed that the effect of global cognition on sleep duration (standardized β = −0.106; 95% CI: −0.116, −0.096; *P* < 0.001; Wave 1 to 2) was larger than the effect of sleep duration on global cognition (standardized β = −0.066; 95% CI: −0.073, −0.059; *P* < 0.001; Wave 1 to 2). Therefore, global cognition appeared to be the main driver in this reciprocal association.

### Sensitivity Analysis

The two unidirectional associations were examined separately by Cox regression and the GEE approach. Cox regression analysis showed that individuals with lower global cognition scores at baseline were more likely to sleep <4 h or >10 h in the follow-up waves (Supplementary Table 6 and Supplementary Figure 2). In the GEE analysis, short or long baseline sleep duration was cross-sectionally associated with lower baseline global cognition. However, baseline sleep duration did not significantly accelerate the decline rate of global cognition during the four waves (Supplementary Table 7).

A forest plot was generated to compare the standardized beta coefficients of the two effects (sleep duration on global cognition and vice versa). The subgroups were based on three different cut-off points for moderate sleep duration. The midpoints of the blue bars were always on the left of those of the red bars, which indicated that the negative effect of global cognition on sleep duration was stronger than the reverse relationship (Figure 4).

The results were materially unchanged in cross-lagged models that included only Wave 1 and Wave 3. Overall, sleep duration and global cognition in the two waves were reciprocally associated with each other, and the effect of global cognition on sleep duration was stronger than the reverse effect (Figure 4 and Supplementary Tables 8, 9). We later excluded three individuals with missing data on baseline demographic and health covariates. After adjustments were made for 16 covariates, the effect size decreased. The negative effect of global cognition on sleep duration remained stronger than the reverse effect in all subgroups (Supplementary Table 10).

The results of the evaluations of the bidirectional relationship between short sleep duration and global cognition and between long sleep duration and global cognition were similar. After individuals with long sleep duration in any of the four waves were excluded, lower global cognition in one wave was associated with short sleep duration in the next wave. The effect of global cognition on short sleep duration was stronger than that of short sleep duration on global cognition (Supplementary Table 11). Additionally, the effect of global cognition on long sleep duration was stronger than that of long sleep duration on global cognition (Supplementary Table 12). Among the 7,984 participants followed up in the four waves, 96 reported a memory-related problems at baseline. The results were similar after exclusion of the 96 participants (Supplementary Table 13).

## Discussion

Using data from a population-based and cognitively healthy sample, we explored the bidirectional association between sleep duration and cognitive function. Our study demonstrated that short or long sleep duration was associated with lower cognition cross-sectionally and longitudinally. Worse sleep duration was associated with lower cognitive function in the next wave, and lower cognition in the previous wave was associated with worse sleep duration in the subsequent wave. Overall, the effect of cognitive function had a greater influence on subsequent sleep duration than the reverse.

Our bidirectional association included the effect of cognition on sleep duration. Previous studies on the effect of cognition on sleep duration are rare, and most of these studies are not population-based. Two studies reported the effect of the cognitive decrement z score over 15 years on later sleep disturbance (Yaffe et al., 2007; West et al., 2021). The significant outcomes included sleep efficiency, sleep fragmentation, sleep latency, and time awake after sleep onset. However, no association was found for sleep duration. Our studies differed in terms of their sample sizes, follow-up durations, and statistical methods used, which would underlie the conflicting results. They also did not view sleep duration as a categorical variable. Several studies have examined the sleep architecture of people at different stages of dementia. Individuals with AD or MCI tend to have excessive daytime sleepiness, insomnia, and sleep-disordered breathing (Guarnieri et al., 2012). Whether sleep duration is a precursor to cognitive decline remains controversial (Leng and Yaffe, 2020; Ma et al., 2020). Using latent growth models and GEE, we found that only short or long sleep duration was cross-sectionally associated with lower cognition at baseline. Baseline sleep duration did not significantly accelerate cognitive decline during a 7-year follow-up. Ma et al. (2020) also examined the association between baseline sleep duration and 4-year cognition in the CHARLS cohort. After controlling for confounders, they found that <4 h and >10 h of sleep at baseline was associated with a faster decline in cognition, while other sleep duration categories would not accelerate cognitive decline (Xie et al., 2019). However, their *P*-value was relatively high (approximately 0.03). Based on the characteristics of our study sample and the separate latent growth models, the proportion of people with a “too long” or “too short” sleep duration, over 90% of whom were short sleepers, tended to increase over time. This shift was due to the average sleep duration of the population declining during aging. In the separate model, the cognitive score also declined over time, which was consistent with the common situation. In the combined latent growth models, we found trends between the slope of the sleep duration value and other trajectories. In other words, a higher worsening rate of sleep duration was associated with a steeper decline in cognitive function. Both the combined latent growth models and Cox regression showed that people with lower cognition at baseline tended to develop worse cognition in the follow-up wave. However, the combined latent growth models were only marginally significant. The effect sizes of the above associations were small due to the small value of the slope of the sleep duration. Moreover, sleep duration and cognitive function were significantly associated with each other by the “intercept.” They were associated with each other at least at baseline and correlated with each other cross-sectionally in other waves, which was widely recognized and reported in our previous study using the CHARLS cohort (Hua et al., 2020a). In conclusion, the combined latent growth models showed a bidirectional association between sleep duration and cognitive function. Our main conclusions were derived from the cross-lagged models. When comparing the effect of sleep duration at one wave on cognitive function at the next wave versus the reverse, the standardized regression coefficient of the global-cognition-to-sleep-duration value was larger. Sensitivity analysis based on different models, cut-off points, and subgroups, showed similar results.

Several mechanisms may explain the impact of sleep duration on cognition. Short sleep duration correlates with many pathologies that lead to lower cognition, such as impaired β-amyloid (Aβ) clearance, pathological tau, impaired synaptic plasticity, atrophy of the cortex, and circadian rhythm disturbances (Yaffe et al., 2014; Malkani and Zee, 2018; Xu et al., 2020). Long sleep duration is also associated with sleep fragmentation and chronic inflammation, which are linked to lower cognition (Lin et al., 2018; Wilcox et al., 2020). A deviation from a 7-h optimal sleep duration was longitudinally associated with cortical atrophy (Spira et al., 2016).

Three AD pathological hallmarks, including Aβ and tau protein accumulation and neurodegeneration, could explain our bidirectional relationship (Van Egroo et al., 2019). Numerous studies have reported that these three markers increase after sleep deprivation or circadian rhythm disruption and lead to cognitive decline, the progression from MCI to AD, and the risk of dementia. These markers also affect sleep structures, including sleep duration. Aβ and tau protein accumulation may start before detectable dementia or at the beginning of cognitive decline. These markers are deposited in areas of the brain that impede sleep-wake regulation. Mice with more Aβ had a 25% shorter sleep duration in the morning (when mice sleep most) (Chemelli et al., 1999). In a transgenic amyloid precursor protein (APP) mouse model, changes in the sleep-wake cycle followed the emergence of Aβ plaques. And the elimination of Aβ deposits by active immunization could normalize the sleep-wake cycle (Roh et al., 2012). However, whether reducing the Aβ burden would improve sleep is not known. A human study found that a higher level of tau in cerebrospinal fluid was associated with lower sleep quality, as reflected by the Pittsburg Sleep Quality Index (PSQI), after 3 years (Fjell et al., 2018). However, there was an interaction between Aβ and tau. A tau knock-in mouse model also showed decreased sleep duration and impaired sleep (Jyoti et al., 2015). Neurodegeneration causes neuronal and synaptic loss in brain regions and networks involved in the control of sleep (Holth et al., 2017). Decreasing cholinergic neurons in sleep-regulating centers, such as the basal forebrain and lateral hypothalamus, alters the sleep-wake cycle (Wilson et al., 2013). Increased or decreased sleep duration and lower cognitive function may also be the result of neurodegeneration.

The foremost strength of our study is its extension of the current understanding from unidirectional to bidirectional and from cross-sectional to longitudinal. Using cross-lagged models, we first investigated associations between two factors simultaneously and found that cognitive function might be the dominant factor. Other advantages include its up-to-date data, a large number of participants, and nationally representative cohort.

Our study also had several limitations. First, the harmonized cognitive test in CHARLS is not a test that is used worldwide, such as the mini-mental status examination (MMSE). Some cognitive domains in the MMSE were not measured, including naming and complex commands. Thus, our cognitive tests might not reflect the real capacity of cognitive performance. We were also unable to find a cut-off point for dementia. Nevertheless, our test is widely used and has a high correlation with the MMSE (Meng et al., 2019). Many articles have demonstrated its reliability and validity (Li et al., 2018; Hua et al., 2020a). Certain cognitive abilities, such as episodic memory, visuospatial abilities, executive function, and processing speed, decline with age. While some abilities, such as recognition memory, language, and crystallized intelligence, can remain stable with age (Harada et al., 2013). The majority of cognitive domains in our study would decrease with age. And, the slope of cognitive ability was significantly negative in our sample, suggesting that our test was sensitive to at least some degree of cognitive aging. Future studies could assess cognitive ability by multiple tests and model this construct as a latent variable. Second, the CHARLS recorded sleep duration using subjective, rather than objective, measurements (such as polysomnography). We could not determine the specific type of sleep disturbance or sleep architecture participating in the bidirectional association. However, the difference between self-reported sleep duration and the objective is smaller in Chinese individuals (49 min, 95% CI: 37–61 min) than in individuals from other ethnicities (Jackson et al., 2018). Subjective sleep duration is more utilized and applicable in epidemiologic studies. Third, people with sleep durations that were too short or too long were classified into one category. As approximately 90% of the subjects were short sleepers, the effect of long sleep duration might be hidden. Fourth, those subjects who remained in the longitudinal study were healthier than those excluded. Exclusion of less healthy participants could result in an underrepresentation of individuals with poorer cognitive ability or sleep function. And thus, it potentially ceased to represent the original study group and resulted in an underestimate of the associations between the variables.

## Conclusion

Our findings support a bidirectional relationship between sleep duration and cognitive function. We hypothesize that short or long sleep duration is not only a lifestyle risk factor but also a marker of cognition-related neurodegenerative progression. The cognitive function might have already declined in people with short or long sleep durations. More attention should be given to the cognitive function of these individuals. The interlinkage of cognitive function and subsequent sleep duration is noteworthy and may have public health implications, raising the possibility that initiatives of cognitive screening and cognitive training among older people with short or long sleep duration may prevent deterioration in sleep duration and cognitive function in later life.

## Data Availability Statement

The data analyzed in this study was obtained from the China Health and Retirement Longitudinal Study (CHARLS). Restrictions apply: researchers should contact the CHARLS office to get the access to use it (http://charls.pku.edu.cn/zh-CN). Requests to access these data can also be directed to Hongpeng Sun (hpsun@suda.edu.cn).

## Ethics Statement

The studies involving human participants were reviewed and approved by the Institutional Review Board at Peking University. The IRB approval number for the main household survey, including anthropometrics, is IRB00001052-11015; the IRB approval number for biomarker collection is IRB00001052-11014. The patients/participants provided their written informed consent to participate in this study.

## Author Contributions

JH contributed to the conception and design of the study. JH and YS organized the database. JH and HS performed the statistical analysis. JH and SZ wrote the first draft of the manuscript. QF and XT reviewed the manuscript. All authors approved the final version of the manuscript.

## Conflict of Interest

The authors declare that the research was conducted in the absence of any commercial or financial relationships that could be construed as a potential conflict of interest.

## Publisher’s Note

All claims expressed in this article are solely those of the authors and do not necessarily represent those of their affiliated organizations, or those of the publisher, the editors and the reviewers. Any product that may be evaluated in this article, or claim that may be made by its manufacturer, is not guaranteed or endorsed by the publisher.
