# Supplementary material for: Exploring the Bidirectional Associations Between Short or Long Sleep Duration and Lower Cognitive Function: A 7-Year Cohort Study in China
Source: Front Aging Neurosci. 2021 Oct 6;13:727763. doi: 10.3389/fnagi.2021.727763 (PMC8528020; doi:10.3389/fnagi.2021.727763)
Supplement: Supplementary file 2 [file Data_Sheet_2.docx]

# Supplementary Materials

# Text

## Supplementary Text 1. Ethics approval

Ethical approval for all the CHARLS waves was granted from the Institutional Review Board at Peking University. The IRB approval number for the main household survey, including anthropometrics, is IRB00001052-11015; the IRB approval number for biomarker collection is IRB00001052-11014. During the fieldwork, each respondent who agreed to participate in the survey was asked to sign two copies of informed consent; one copy was kept in the CHARLS office, which was also scanned and saved in PDF format. Four separate consents were obtained: one for the main fieldwork, one for the non-blood biomarkers and one for the taking of the blood samples, and another for storage of blood for future analyses.

## Supplementary Text 2. Cognitive assessment methods

The episodic memory test was composed of immediate and delayed (five minutes later) recall for 10 unrelated words. The test score was the average number of immediate and delayed-recall words and ranged from 0 to 10. In the figure drawing test, which was used to assess visuospatial construction, the participants were shown a picture and asked to redraw it; one point was given for success. The Telephone Interview of Cognitive Status (TICS) in CHARLS was based on selected questions in the mini-mental status examination (MMSE) and comprised ten mental questions: a serial subtraction of 7 from 100 for five times; the date (day, month, year); the day of the week; and the season. This test reflects participants’ attention, orientation and calculation abilities. This score ranged from 0 to 10.

# Tables

## Supplementary Table 1. Comparison of baseline characteristics between participants included (n=7984) and excluded due to loss to follow-up (n=6997)^a^

| **Characteristic** | **Included (n=7984)** | **Excluded**  **(n=6997)** | **P for difference^*^** |
| --- | --- | --- | --- |
| Age (years) | 57.7 ± 8.4 | 61.3 ± 10.3 | <0.001 |
| Number of IADLs | 0.1 ± 0.5 | 0.3 ± 0.9 | <0.001 |
| Global cognition scores | 11.1 ± 4.0 | 9.7 ± 4.6 | <0.001 |
| Sleep duration (hour) | 6.5 ± 1.8 | 6.3 ± 2.0 | <0.001 |
| Male | 3858 (48.4) | 3301 (47.2) | 0.169 |
| Living area |  |  | <0.001 |
| Urban | 1533 (19.3) | 1997 (28.6) |  |
| Rural | 6442 (80.8) | 4989 (71.4) |  |
| Education |  |  | <0.001 |
| Illiterate | 1669 (20.9) | 2349 (33.6) |  |
| Primary school | 3373 (42.3) | 2567 (36.7) |  |
| Middle school | 1912 (24.0) | 1191 (17.0) |  |
| High school and above | 1027 (12.9) | 883 (12.6) |  |
| Married | 7298 (91.4) | 5860 (83.7) | 0.008 |
| Current smoking | 3115 (39.3) | 2779 (39.7) | 0.381 |
| Current drink | 2073 (26.0) | 1686 (24.1) | 0.009 |
| Hypertension | 1823 (22.8) | 1834 (26.2) | <0.001 |
| Dyslipidemia | 758 (9.5) | 623 (8.9) | 0.213 |
| Diabetes | 449 (5.6) | 411 (5.9) | 0.511 |
| Lung disease | 739 (9.3) | 803 (11.5) | <0.001 |
| Cancer | 68 (0.9) | 76 (1.1) | 0.142 |
| Heart problems | 920 (11.5) | 903 (13.0) | 0.008 |
| Stroke | 129 (1.6) | 182 (2.6) | <0.001 |
| Depression | 1598 (20.0) | 1634 (23.4) | <0.001 |

^a^The results were presented as mean ± SD, or n (%). For each characteristic, missing data was less than 20.

^*^The differences between participants included and excluded were tested using the *t*-test or chi-square test.

## Supplementary Table 2. Intercept and slope estimate of sleep duration and global cognition using the separate linear latent growth models^*^

| Models | Intercept (SE) | Intercept Variance (SE) | Slope (SE) | Slope Variance (SE) |
| --- | --- | --- | --- | --- |
| Trajectory of sleep duration | 0.071 (0.003) | 0.023 (0.002) | 0.006 (0.001) | <0.001 (<0.001) |
| Trajectory of global cognition | 11.362 (0.041) | 8.879 (0.224) | -0.201(0.006) | 0.280 (0.007) |

^*^P<0.001 for all values. All coefficients were unstandardized.

## Supplementary Table 3. Intercept and slope estimate of sleep duration and global cognition using the separate quadratic latent growth models

|  | Unstandardized coefficient (SE) | P value |
| --- | --- | --- |
| Sleep duration^a^ |  |  |
| Intercept | 0.073 (0.003) | <0.001 |
| Intercept variance | 0.021 (0.002) | <0.001 |
| Linear slope | 0.004 (0.002) | 0.021 |
| Linear slope variance | 0.002 (0.001) | 0.015 |
| Quadratic slope | 0.000 (0.000) | 0.304 |
| Quadratic slope variance | 0.000 (0.000) | <0.001 |
| Global cognition |  |  |
| Intercept | 11.086 (0.044) | <0.001 |
| Intercept variance | 10.086 (0.042) | <0.001 |
| Linear slope | 0.093 (0.019) | <0.001 |
| Linear slope variance | 0.032 (0.109) | 0.003 |
| Quadratic slope | -0.042 (0.003) | <0.001 |
| Quadratic slope variance | 0.001 (0.002) | 0.577 |

^a^Goodness of fit: CFI, 1.000; TLI, 0.997; RMSEA, 0.026.

^b^Goodness of fit: CFI, 1.000; TLI, 1.002; RMSEA, 0.000.

## Supplementary Table 4. Unstandardized coefficients of confounders on trajectories of sleep duration and cognitive function

|  | Sleep duration  baseline | Sleep duration  slope | Global cognition  baseline | Global cognition  slope |
| --- | --- | --- | --- | --- |
| Age | 0.003^***^ | <0.001^**^ | -0.102^***^ | -0.012^***^ |
| Sex (male) | -0.040^***^ | -0.005^***^ | 1.839^***^ | 0.001 |
| Living area (Urban) | -0.028^***^ | -0.003^*^ | 2.671^***^ | 0.039^*^ |

^*^P<0.05, ^**^P<0.01, ^***^P<0.001.

## Supplementary Table 5. Confounders in cross-lagged models which across all four waves

|  | Age | Sex (female) | Living area (urban) |
| --- | --- | --- | --- |
| Standardized |  |  |  |
| Sleep duration Wave 1 | 0.164^***^ | 0.122^***^ | -0.104^***^ |
| Sleep duration Wave 2 | 0.037 | 0.085^***^ | 0.017 |
| Sleep duration Wave 3 | 0.030 | 0.085^***^ | -0.020 |
| Sleep duration Wave 4 | 0.032 | -0.007 | -0.022 |
| Global cognition Wave 1 | -0.231^***^ | -0.228^***^ | 0.271^***^ |
| Global cognition Wave 2 | -0.115^***^ | -0.097^***^ | 0.110^***^ |
| Global cognition Wave 3 | -0.070^***^ | -0.008 | 0.031^**^ |
| Global cognition Wave 4 | -0.122^***^ | -0.026^**^ | -0.059^***^ |
| Unstandardized |  |  |  |
| Sleep duration Wave 1 | 0.020^***^ | 0.250^***^ | -0.270^***^ |
| Sleep duration Wave 2 | 0.006 | 0.222^***^ | 0.056 |
| Sleep duration Wave 3 | 0.005 | 0.246^***^ | -0.074 |
| Sleep duration Wave 4 | 0.006 | -0.021 | -0.083 |
| Global cognition Wave 1 | -0.110^***^ | -1.820^***^ | 2.740^***^ |
| Global cognition Wave 2 | -0.053^***^ | -0.743^***^ | 1.072^***^ |
| Global cognition Wave 3 | -0.033^***^ | -0.055^***^ | 0.309^***^ |
| Global cognition Wave 4 | -0.072^***^ | 0.256^**^ | 0.744^***^ |

^**^P<0.01, ^***^P<0.001.

## Supplementary Table 6. Association between baseline cognitive function and new-onset events

| New-onset events^a^ | Group 1^b^ | Group 2 | Group 3 | Group 4 |
| --- | --- | --- | --- | --- |
| Cases n (%) | 169 (8.8) | 216 (12.5) | 294 (16.3) | 553 (28.3) |
| Model 0^d^ | 1 (Ref.) | 1.444 (1.180-1.765) ^c^ | 1.386 (1.261-1.524) | 1.517 (1.432-1.607) |
| Model 1^e^ | 1 (Ref.) | 1.405 (1.144-1.724) | 1.299 (1.178-1.433) | 1.390 (1.304-1.482) |

^a^New-onset event means slept less than 4 h or longer than 10 h in any of the wave between Wave 2 to Wave 4.

^b^Global cognition ranges: group 1, 14.5 to 21; group 2, 12.0 to 14; group 3, 9.0 to 11.5; group 4, 0 to 8.5.

^c^Hazard ratios (95% CI), for all such values.

^d^Model 0: unadjusted.

^e^Model 1: adjusted for age, sex and living area.

## Supplementary Table 7. Longitudinal cognitive changes by sleep category using GEE model

|  | Sleep category  β (SE) | Time  β (SE) | Sleep category*time  β (SE) |
| --- | --- | --- | --- |
| Model 0 |  |  |  |
| <4 h or > 10 h | -2.336 (0.156) ^***^ | -0.206 (0.007) ^***^ | -0.037 (0.025) |
| <5 h or > 9 h | -1.637 (0.1060^***^ | -0.202 (0.007) ^***^ | -0.023 (0.017) |
| <6 h or > 8 h | -1.783 (0.087) ^***^ | -0.196 (0.008) ^***^ | -0.012 (0.013) |
| Model 1 |  |  |  |
| <4 h or > 10 h | -1.160 (0.143) ^***^ | -0.206 (0.007) ^***^ | -0.036 (0.025) |
| <5 h or > 9 h | -1.050 (0.097) ^***^ | -0.202 (0.007) ^***^ | -0.023 (0.017) |
| <6 h or > 8 h | -0.736 (0.080) ^***^ | -0.196 (0.008) ^***^ | -0.012 (0.014) |

*P<0.001.

Model 0: Crude model.

Model 1: Adjusted for age, sex and living area.

## Supplementary Table 8. Cross-lagged models across Wave1 and Wave3^a^

|  | Unstandardized β (SE) | Standardized β (SE) |
| --- | --- | --- |
| Sleep duration to global cognition | -0.359 (0.059) | -0.086 (0.014) |
| Global cognition to sleep duration | -0.026 (0.005) | -0.103 (0.019) |

^a^This model included Wave 1 and Wave 3. 4 h and 10 h were the cut-off points. AFI=1.000, TLI=1.000, RMSEA=0.002.

## Supplementary Table 9. Confounders in cross-lagged models across Wave 1 and Wave 3^a^

|  | Age | Sex (male) | Living area (urban) |
| --- | --- | --- | --- |
| Standardized |  |  |  |
| Sleep duration Wave 1 | -0.050*** | 0.002 | -0.026* |
| Sleep duration Wave 3 | 0.037** | -0.005 | -0.035** |
| Global cognition Wave 1 | -0.253*** | 0.236*** | 0.269*** |
| Global cognition Wave 3 | -0.191*** | 0.090*** | 0.116*** |
| Unstandardized |  |  |  |
| Sleep duration Wave 1 | -0.006*** | 0.005 | -0.065* |
| Sleep duration Wave 3 | 0.004** | -0.010 | -0.089** |
| Global cognition Wave 1 | -03119*** | 1.936*** | 2.774*** |
| Global cognition Wave 3 | -0.094*** | 0.770*** | 1.265*** |

^*^P<0.05, ^**^P<0.01, ^***^P<0.001.

^a^This model included Wave 1 and Wave 3.

## Supplementary Table 10. Cross-lagged models including Wave 1 and Wave 3 with adjustments for demographic and health covariates^a^

|  | Unstandardized β (SE) | Standardized β (SE) |
| --- | --- | --- |
| <4 h or >10 h vs. 4-10 h (n=10015) |  |  |
| Sleep duration to global cognition | -0.256 (0.057)^*^ | -0.064 (0.014) |
| Global cognition to sleep duration | -0.022 (0.005) | -0.085 (0.020) |
| <5 h or >9h vs. 5-9 h (n=9932) |  |  |
| Sleep duration to global cognition | -0.220 (0.043) | -0.055 (0.011) |
| Global cognition to sleep duration | -0.017 (0.004) | -0.066 (0.016) |
| <6 h or >8 h vs. 6-8h (n=9682) |  |  |
| Sleep duration to global cognition | -0.155 (-0.039) | -0.038 (0.010) |
| Global cognition to sleep duration | -0.016 (0.004) | -0.064 (0.015) |

^a^Adjusted for age, sex, education, marital status, living area, current smoking, current drinking, hypertension, dyslipidemia, diabetes, cancer, lung diseases, heart problems, stroke, depression, and number of IADLs. Three individuals with missing data on baseline covariates were excluded. For “4-10” group, 6 individuals with “excessive change” were excluded. For the “5-9” group, 89 individuals with “excessive change” were excluded. For the “6-8” group, 339 individuals with “excessive change” were excluded.

^*^P<0.001 for all values.

## Supplementary Table 11. Cross-lagged models across four waves, excluding participants with long sleep duration in any of the four waves^*^

|  | Wave 1 to 2 | Wave 2 to 3 | Wave 3 to 4 |
| --- | --- | --- | --- |
| <4 h vs. 4-10 h (n=7754) | | | |
| Sleep duration to global cognition | -0.061 (0.007)^a^ | -0.077 (0.009) | -0.070 (0.008) |
| Global cognition to sleep duration | -0.096 (0.011) | -0.081 (0.009) | -0.079 (0.009) |
| <5 h vs. 5-9h (n=6963) | | | |
| Sleep duration to global cognition | -0.051 (0.006) | -0.062 (0.007) | -0.055 (0.006) |
| Global cognition to sleep duration | -0.082 (0.009) | -0.071 (0.008) | -0.070 (0.008) |
| <6 h vs. 6-8 h (n=6141) | | | |
| Sleep duration to global cognition | -0.041 (0.006) | -0.048 (0.007) | -0.042 (0.006) |
| Global cognition to sleep duration | -0.066 (0.009) | -0.058 (0.008) | -0.058 (0.008) |

^*^Adjusted for age, sex, and living area. For the “4-10” group, 230 individuals with long sleep duration were excluded, and 1637 of the included individuals had short sleep duration in any of the four waves. For the “5-9” group, 1021 individuals with long sleep duration were excluded, and 3025 individuals had short sleep duration. For the “6-8” group, 1841 individuals with long sleep duration were excluded, and 4860 individuals had short sleep duration.

^a^Standardized β (SE) for all such values. P<0.001 for all values.

## Supplementary Table 12. Cross-lagged models across four waves, excluding participants with short sleep duration in any of the four waves^*^

|  | Wave 1 to 2 | Wave 2 to 3 | Wave 3 to 4 |
| --- | --- | --- | --- |
| >10 h vs. 4-10 h (n=6367) | | | |
| Sleep duration to global cognition | -0.263 (0.021)^a^ | -0.254 (0.021) | -0.242 (0.015) |
| Global cognition to sleep duration | -0.270 (0.024) | -0.294 (0.022) | -0.255 (0.022) |
| >9 h vs. 5-9 h (n=6963) | | | |
| Sleep duration to global cognition | -0.077 (0.012) | -0.083 (0.013) | -0.068 (0.010) |
| Global cognition to sleep duration | -0.116 (0.016) | -0.105 (0.015) | -0.108 (0.015) |
| >8 h vs. 6-8 h (n=6141) | | | |
| Sleep duration to global cognition | -0.059 (0.011) | -0.064 (0.012) | -0.053 (0.010) |
| Global cognition to sleep duration | -0.130 (0.015) | -0.118 (0.014) | -0.123 (0.014) |

^*^Adjusted for age, sex, and living area. For the “4-10” group, 1637 individuals with long sleep duration were excluded. 230 of the included individuals had long sleep duration in any of the four waves. For the “5-9” group, 3058 individuals with short sleep duration were excluded, and 1021 individuals had long sleep duration. For the “6-8” group, 1617 individuals with short sleep duration were excluded, and 1843 individuals had long sleep duration.

^a^Standardized β (SE) for all such values. P<0.001 for all values.

## Supplementary Table 13. Cross-lagged models across four waves, excluding 96 participants with memory related problems at baseline*

|  | Wave 1 to 2 | Wave 2 to 3 | Wave 3 to 4 |
| --- | --- | --- | --- |
| <4h or >10h vs. 4-10h (n=7888)^a^ | | | |
| Sleep duration to global cognition | -0.065 (0007) ^b^ | -0.080 (0.008) | -0.072 (0.008) |
| Global cognition to sleep duration | -0.104 (0.011) | -0.090 (0.009) | -0.089 (0.009) |
| <5h or >9h vs. 5-9h (n=7784) | | | |
| Sleep duration to global cognition | -0.056 (0.006) | -0.064 (0.007) | -0.071 (0.005) |
| Global cognition to sleep duration | -0.099 (0.009) | -0.090 (0.008) | -0.089 (0.008) |
| <6 h or >8h vs. 6-8h (n=7473) |  |  |  |
| Sleep duration to global cognition | -0.065 (0.007) | -0.079 (0.009) | -0.071 (0.008) |
| Global cognition to sleep duration | -0.104 (0.011) | -0.090 (0.009) | -0.089 (0.009) |

^a^For “4-10” group, none of the participants exhibited “excessive change”. For “5-9” group, 4 individuals with “excessive change” were excluded. For “6-8” group, 415 individuals with “excessive change” were excluded.

^b^Standardized β (SE) for all such values. P<0.001 for all values.

# Figures

## Supplementary Figure 1. Association between changes in sleep duration and cognitive function.

Supplementary Figure 1(a) demonstrated that a deviation from the optimal sleep duration was detrimental to cognitive function. We defined “excessive change” as those who slept short in the previous wave and slept long in the next wave, or vice versa. “Excessive change” also harmed cognitive function. Supplementary Figure 1(b) and 2(c) showed the results of a previous study and our previous findings*(Hua et al., 2020; Zhu et al., 2020)*. Therefore, we hypothesized an association between changes in sleep duration and cognitive function: changes from short or long sleep duration to moderate sleep duration benefits cognition, deviations from the moderate sleep duration and “excessive change” harms cognition.


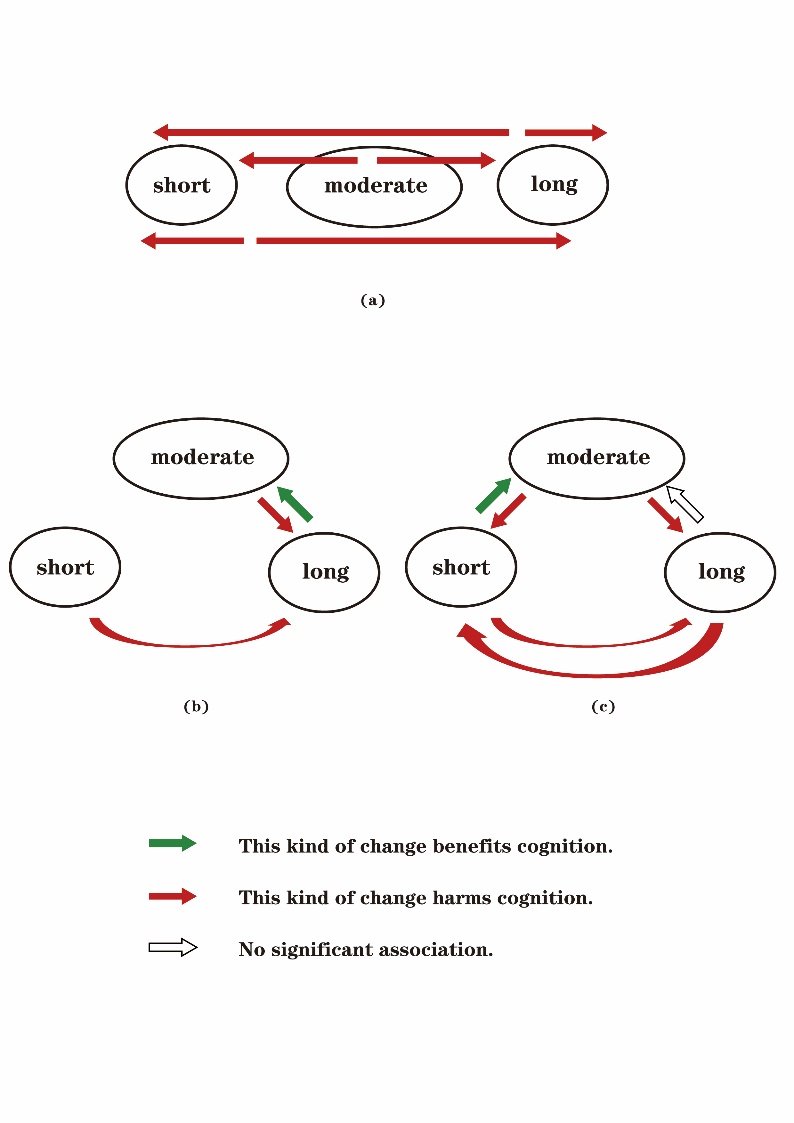


## **
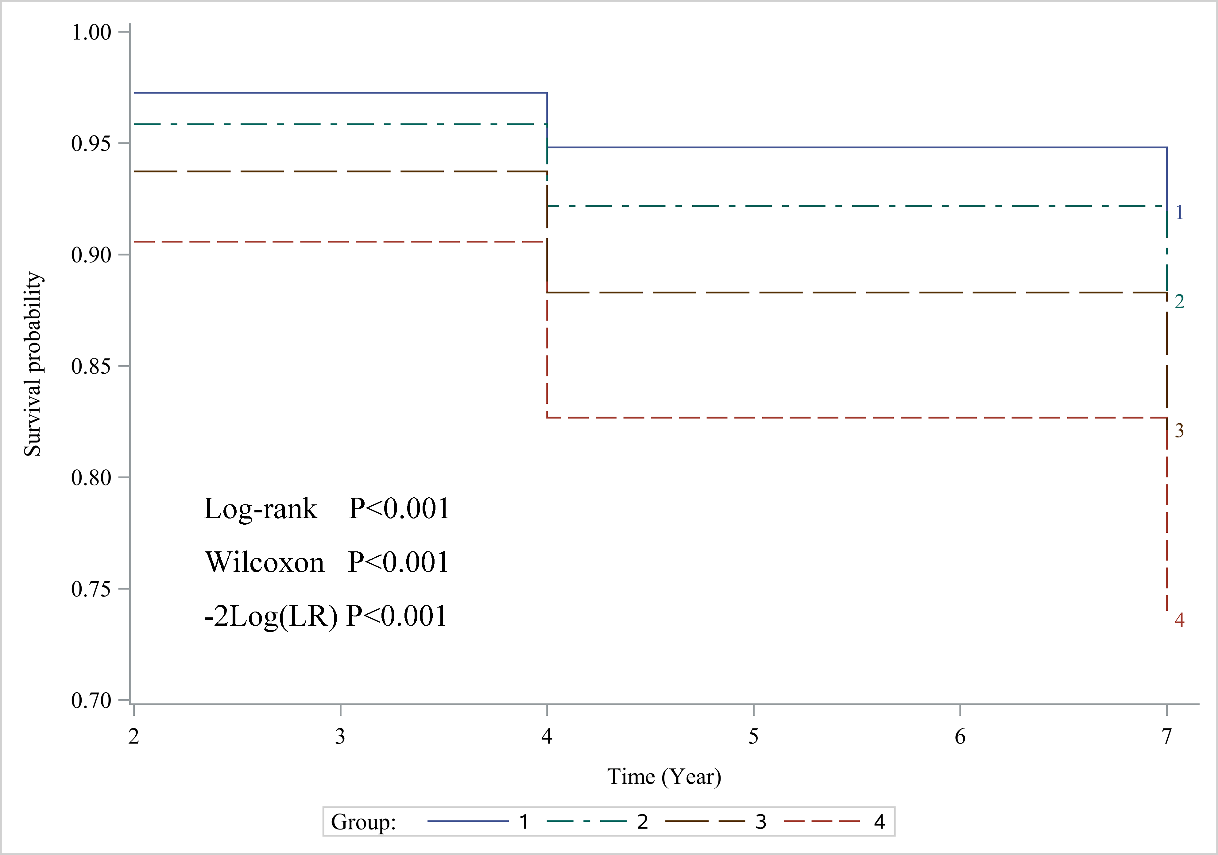
Supplementary Figure 2. Kaplan-Meier curves for the cumulative risk of events in quantiles.** Global cognition score ranges: group 1, 14.5 to 21; group 2, 12 to 14; group 3, 9 to 11.5; group 4, 0 to 8.5.

## **Supplementary Figure 3. Forest plot based on different cut-off points and models, retaining participants with “excessive change”.**


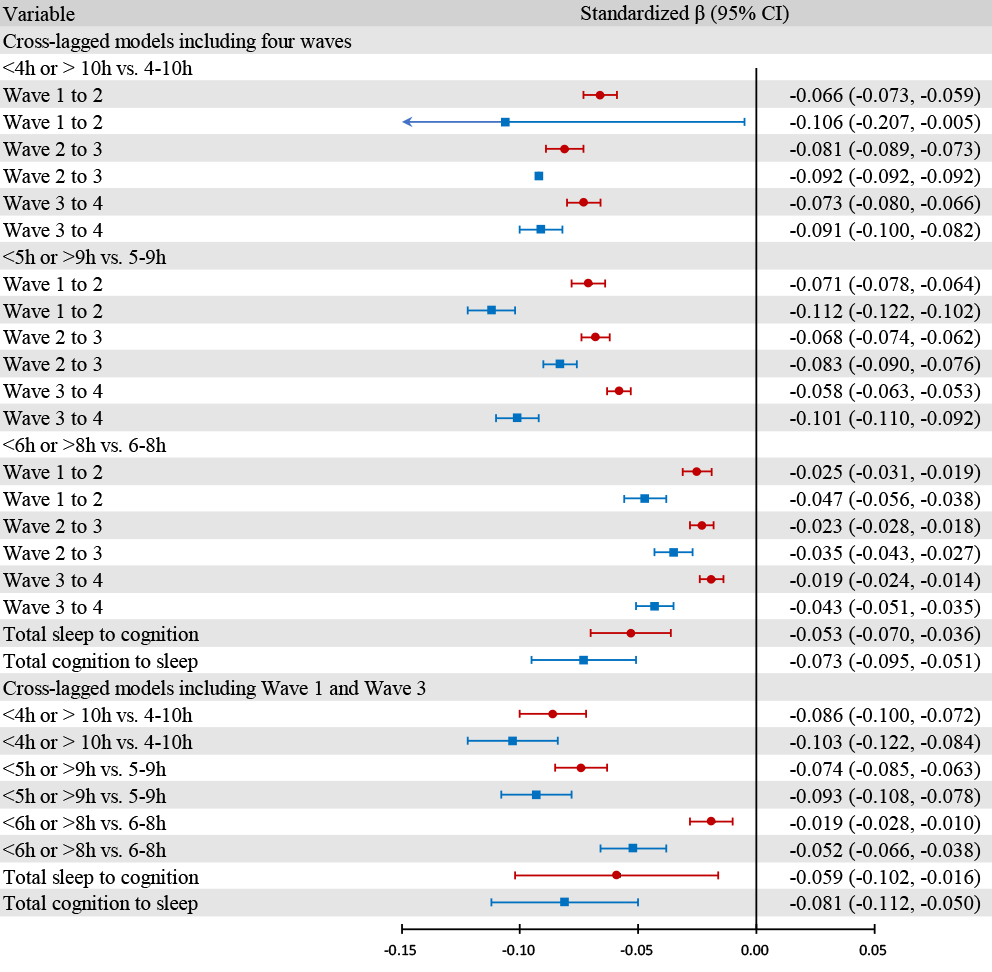


# References

Hua, J., Sun, H., and Shen, Y. (2020). Improvement in sleep duration was associated with higher cognitive function: a new association. *Aging (Albany NY)* 12(20)**,** 20623-20644. doi: 10.18632/aging.103948.

Zhu, Q., Fan, H., Zhang, X., Ji, C., and Xia, Y. (2020). Changes in sleep duration and 3-year risk of mild cognitive impairment in Chinese older adults. *Aging (Albany NY)* 12(1)**,** 309-317. doi: 10.18632/aging.102616.
